# Assessment of the Patient’s Emotional Response with the RobHand Rehabilitation Platform: A Case Series Study

**DOI:** 10.3390/jcm11154442

**Published:** 2022-07-30

**Authors:** Ana Cisnal, Victor Moreno-SanJuan, Juan Carlos Fraile, Javier P. Turiel, Eusebio de-la-Fuente, Guillermo Sánchez-Brizuela

**Affiliations:** Instituto de las Tecnologías Avanzadas de la Producción (ITAP), School of Industrial Engineering, University of Valladolid, Prado de la Magdalena, 47011 Valladolid, Spain; victor.moreno@alumnos.uva.es (V.M.-S.); jcfraile@eii.uva.es (J.C.F.); turiel@eii.uva.es (J.P.T.); efuente@uva.es (E.d.-l.-F.); guillermo.sanchez.brizuela@uva.es (G.S.-B.)

**Keywords:** neurorehabilitation, rehabilitation robotics, emotional assessment, virtual environments

## Abstract

Cerebrovascular accidents have physical, cognitive and emotional effects. During rehabilitation, the main focus is placed on motor recovery, yet the patient’s emotional state should also be considered. For this reason, validating robotic rehabilitation systems should not only focus on their effectiveness related to the physical recovery but also on the patient’s emotional response. A case series study has been conducted with five stroke patients to assess their emotional response towards therapies using RobHand, a robotic hand rehabilitation platform. Emotional state was evaluated in three dimensions (arousal, valence and dominance) using a computer-based Self-Assessment Manikin (SAM) test. It was verified that the emotions induced by the RobHand platform were successfully distributed in the three-dimensional emotional space. The increase in dominance and the decrease in arousal during sessions reflects that patients had become familiar with the rehabilitation platform, resulting in an increased feeling of control and finding the platform less attractive. The results also reflect that patients found a therapy based on a virtual environment with a realistic scenario more pleasant and attractive.

## 1. Introduction

The possible implications of stroke are not only physical and motor limitations but also cognitive impairment and emotional and social consequences for both the patients and their families. Stroke rehabilitation has mainly focused on the importance of physical recovery, emphasizing the relevance of motor rehabilitation to enable patients to perform daily living activities. Regarding physical recovery as the principal outcome of stroke rehabilitation has led to neglecting the emotional and social negative effects of stroke. Many patients, regardless of their grade of physical recovery, suffer from emotional disorders, such as anxiety, agoraphobias and depression [1].

There is evidence to show that emotional states such as stress, discomfort, boredom or lack of motivation can have an impact on motor learning [2,3,4]. Anxiety, frustration or stress can result in a worsening of motor performance [5], while high levels of arousal can increase it [6,7]. Cognitive states can either increase or decrease human performance [8]: a bored person tends to perform worse than a focused one [3,9].

However, there is no clear evidence of how emotions, feelings and attitudes influence functional recovery after suffering a stroke [10]. Despite this lack of evidence, maintaining a positive attitude towards motor rehabilitation is considered to be an important factor. Undoubtedly, the greater the motivation and the better the attitude, the more likely the patient is to be actively engaged. Many studies have indicated that intensity in rehabilitation is a crucial factor in achieving greater motor recovery [11,12].

During traditional rehabilitation, a major role of the healthcare professional is to maintain the patients’ motivation while undertaking the required physical exercises to recover the motor function of the impaired limbs. An aging population results in an increase in the number of stroke survivals with severe disabilities [13,14], while the healthcare–patient ratio is being reduced [15]. For this reason, interest in robotic rehabilitation platforms has shown significant growth in the last decade.

One of the great benefits of robotic rehabilitation platforms is allowing the healthcare professional to be simultaneously supervising several patients in the so-called robotic gyms. However, this also has the disadvantage of preventing them from spending enough time with patients, as they do during traditional rehabilitation. Thus, healthcare professionals are forced to neglect the emotional care of the patients, which requires time and individual attention.

The evaluation of motor function by using widely known functional tests, such as the Fugl-Meyer Assessment (FMA) or the Action Research Arm Test (ARAT), is essential for determining stroke severity, describing motor recovery and planning treatment. On the other hand, the emotional state of patients typically receives less attention during post-stroke rehabilitation [3,16]. We consider that the latter should also be assessed while undertaking rehabilitation using robotic platforms. Furthermore, the emotional response of the user should be a factor to consider when designing and validating rehabilitation robots.

There are two main approaches for describing emotions: (1) a discrete approach of a universal set of basic emotions [17] and (2) a dimensional approach using two or more dimensions for a major emotion, so they can be combined to describe different emotions [18]. There is no consensus as to which method is the best for assessing emotions. However, previous studies in the field of human–robot interaction (HRI) have used the dimensional approach because of its simplicity in comparison with the discrete one [19].

Emotions can be reported using three dimensions: arousal (sleepy–focused), valence (miserable–happy) and dominance (controlling–controlled) [18]. This three-dimensional approach is also known as the PAD emotional state model (PAD stands for Pleasure, Arousal and Dominance) [20]. More adjective pairs associated with the three emotional dimensions [21] can be found in Table 1. While some studies related to HRI used the three-dimensional approach [4,22], others used a two-dimensional approach, in which emotions are only represented by arousal and valence as independent variables [19,23,24]. However, it has been suggested to replace the often-used two-dimensional model with a three-dimensional model which includes a third dominance axis so that it can not only can evaluate affect/feeling (valence) and cognition/thinking (arousal) but also behavior/acting (dominance) [25].

Emotions are accompanied by a set of somatic responses associated with the autonomic nervous system (ANS) activity and can be determined directly or indirectly. The Self-Assessment Manikin (SAM) test [25] is used to directly assess the three affective dimensions of the patient in response to a wide variety of stimuli using a non-verbal pictorial questionnaire. The circumplex model of affect and the affect grid are used to assess emotions along the dimensions of pleasure and arousal using a single-item scale [18,26]. On the other hand, psychophysiological tools can be used to indirectly measure ANS-related responses to external stimuli [27]. Parameters such as heart rate, skin temperature, respiration rate, galvanic skin response or electrocardiogram have been used to try to indirectly evaluate emotions in HRI studies [28,29,30,31,32,33].

After successfully testing the RobHand (Robot for Hand Rehabilitation) with healthy subjects in terms of usability, comfort and finger kinematics [34,35], we aimed to evaluate the emotional response produced in patients who have suffered a cerebrovascular accident. The research was conducted with five patients, selected by applying the corresponding inclusion/exclusion criteria to assess their emotional state. The emotional state is directly assessed using the SAM test, and the three emotional dimensions are considered: valence, dominance and arousal. The results and discussion of the test outcomes are detailed in the present manuscript.

## 2. Materials and Methods

### 2.1. Participants

A study was performed at the Hospital Clínico Universitario de Valladolid (HCUV), Valladolid, Spain, in order to test and validate the usability of the platform under the passive training modality of RobHand and to evaluate the emotional response of patients when exercising using it.

A total of five patients were involved in the study (Table 2). The medical team was responsible for recruiting patients who were receiving physiotherapy treatment after a stroke. All patients were properly informed by the medical staff, and they gave written consent before starting the study, indicating that they understood the purpose and requirements of the study.

The inclusion criteria were: patients with a diagnosis of cerebral infarction or spontaneous intracerebral hemorrhage that causes significant, but not complete, disabling paresis of an upper limb as a sequel and people with stroke in the chronic and stabilized phase from the ischemic/hemorrhagic event. The exclusion criteria were: patients with zero mobility and cognitive impairment prior to stroke were excluded.

### 2.2. RobHand Rehabilitation Platform

RobHand is a rehabilitation platform for performing passive and active assisted training exercises involving the flexion and extension the metacarpophalangeal (MCP) and proximal interphalangeal (PIP) joints of each hand finger. It is composed of a hand exoskeleton and a software environment.

#### 2.2.1. Mechatronic Device

**Figure 1:**
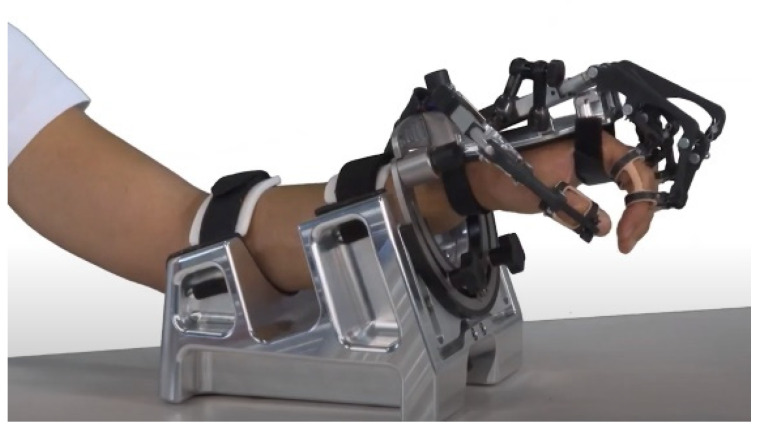
The RobHand hand exoskeleton and the forearm support.

The RobHand exoskeleton (Figure 1) is based on a 4-bar linkage underactuated mechanism that assists the hand opening and closing [34]. MCP and PIP joint movements of each finger are driven by one linear motor. Flexible double-rings, which are first placed on each of the fingers and then clamped to the exoskeleton, ensure easy positioning of the device. The rehabilitation platform integrates a forearm support to mitigate the forces and torques created by the weight of the exoskeleton (610 g).

#### 2.2.2. Passive Training Control

The passive training modality is based on programmed rehabilitation exercises, which involve the repetition of finger flexion and extension movements at three predefined velocities of the MCP joint: low (20°/s), medium (25°/s) and high (35°/s). The types of movement-based exercises available are: (a) flexion and extension of the five hand fingers simultaneously; (b) fingers opening and closing: flexion and extension of hand fingers individually; and (c) flexion and extension of the thumb against index finger (precision grip) or against the four fingers (pinch grip).

**Figure 2:**
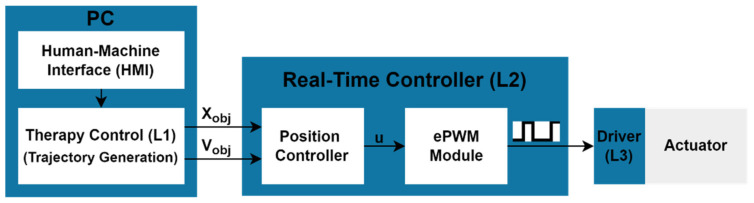
Control schema for the passive mode control.

The open-loop control schema for the passive training is shown in Figure 2. The healthcare professional selects one of these three exercises and introduces its characteristic parameters (opening and closing maximum angles, stop time between open and close movements and speed) using a specific user interface. The therapy control (L1) module and, especially, the trajectory generator sends the target position (x_obj_) and speed (v_obj_) to the real-time controller (L2) during the duration of the exercise according to the introduced parameters. The robot moves the hand fingers to the target position at the specified speed by an open-loop position control implemented in L2. The position controller calculates the control signal (u), which determines the pulse width that the ePWM module must generate in order to move the actuator to the target position at the chosen speed.

**Figure 3:**
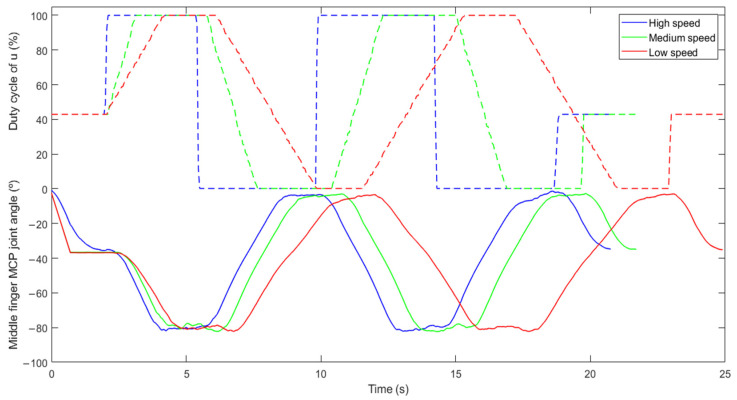
Recorded data of the MCP joint angle of the middle finger (full line) and duty cycle of the applied control signal u (dashed line) for different speeds (high, medium and low) when performing a flexion and extension of the five hand fingers simultaneously (2 repetitions and 2 s pause between closing–opening movement).

In the passive training modality, the hand is continuously moved. During passive movements, patients are instructed to relax their hand, while the motors move their fingers with a comfortable ROM and speed selected by the healthcare professional according to the patient’s residual skill. The MCP joint angle and percentage of duty cycle for the control signal (u) of the middle finger while performing passive therapies are shown in Figure 3.

Figure 3 shows that the duration of the three therapies is not the same even though the number of repetitions (2 repetitions), the pause time between closing and opening movement (2 s), the degrees of opening (2°) and closing (−78°) and the type of therapy (simultaneous flexion and extension of the five hand fingers) are the same. What makes the difference in the overall duration is the chosen velocity: high (blue line), medium (green line) and low (red line). For the high speed, the duty cycle of the control signal (u) changes from the current point to a new point immediately when there is a shift in the target position. Thus, the speed reached by the actuators is the maximum and, consequently, the duration for reaching that new target position is the shortest. On the contrary, the duty cycle of the control signal for the other predefined velocities changes gradually when there is a change in the target position. Figure 3 also shows that the hand exoskeleton allows patients to perform movements according to its range of motion. In this case, the patient is able to perform a hyperextension of his middle finger up to 2° and to perform an extension of 78°.

#### 2.2.3. Software Environment

**Figure 4:**
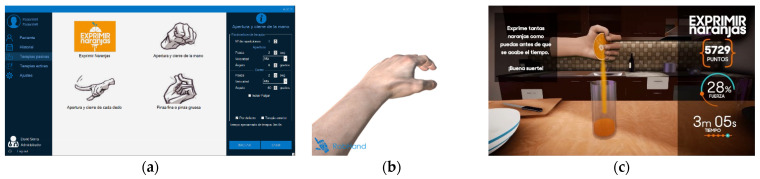
Snapshot of the software environment of RobHand: (**a**) Tab for configuration and selecting passive therapies; (**b**) Virtual reality environment for therapies T1–T3; (**c**) Virtual reality environment for therapy T4.

The software environment allows the healthcare staff to select and configurate therapies according to the patient’s needs (Figure 4a). There are four passive therapies available: (T1) hand flexion/extension therapy, (T2) precision or pinch grips therapy, (T3) individual finger flexion/extension therapy and (T4) squeeze oranges therapy. The configuration parameters (right panel in Figure 4a) are number of repetitions, speed, opening and closing angles and waiting time between opening and closing movements.

The four therapies are based on virtual reality environments; while therapies T1–T3 show just a virtual hand replicating the real movements of the hand exoskeleton (Figure 4b), therapy T4 is based on a virtual kitchen where the user squeezes oranges (Figure 4c).

The relationship between the four available therapies and the type of movement-based exercises and virtual environment used can be found in Table 3.

### 2.3. Self-Assessment Manikin Test

**Figure 5:**
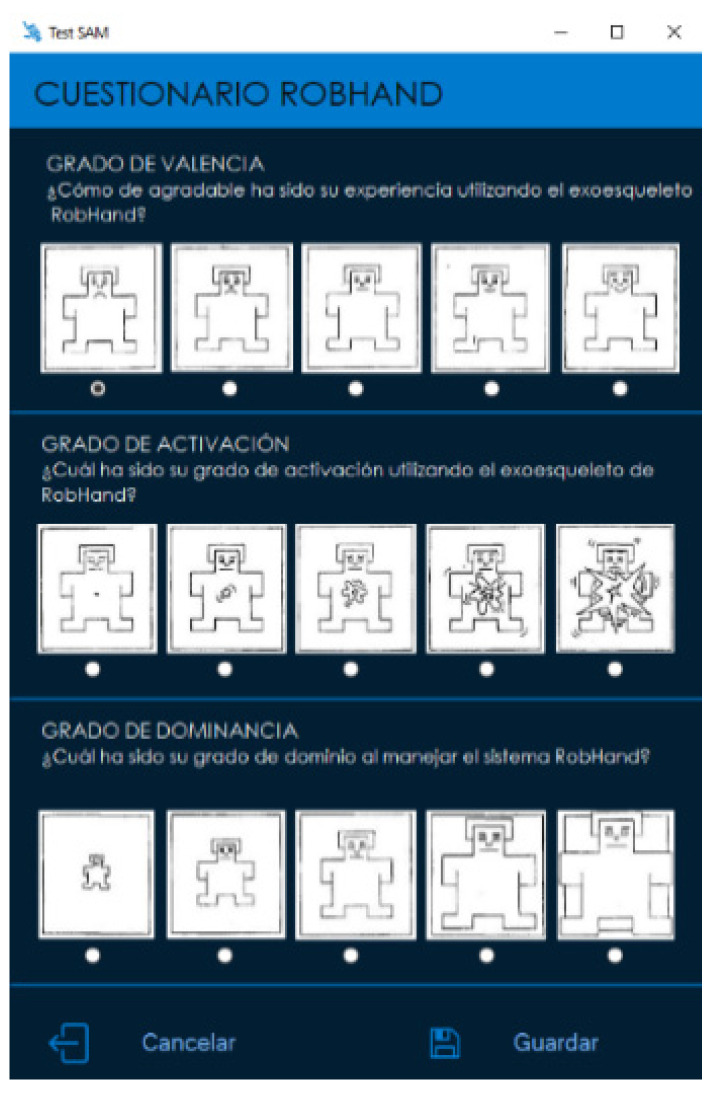
Computer-based SAM test.

The Self-Assessment Manikin (SAM) test was used to subjectively assess the emotional response of the patients. This test is a picture-oriented questionnaire to independently evaluate three emotional dimensions: arousal (i.e., emotional activation), valence (i.e., pleasure) and dominance (i.e., sense of control) [25]. We provided a computer-based SAM test (Figure 5) so that patients could select the answers without any external human intervention that might have otherwise biased their choice.

The SAM evaluation test was used to obtain the ratings (from 1 to 5) in the valence, arousal and dominance dimensions. The higher the rating, the more intense the emotion would be (high pleasure, high arousal and high dominance).

### 2.4. Experimental Setup and Protocol

**Figure 6:**
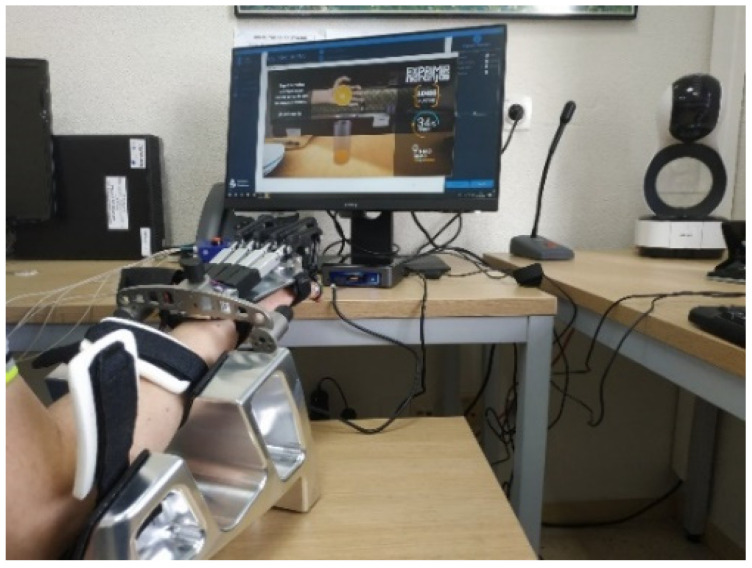
Setup for therapies with RobHand exoskeleton at HCUV hospital.

Patients were seated in a chair wearing the RobHand exoskeleton in their paretic hand (Figure 6). The forearm was placed on an arm rest in a neutral position. For each patient, the linkage-rings of the exoskeleton were adjusted to fit the hand size. The hand was strapped to the device and patients performed hand movements according to the protocol shown in Table 4. Each patient received 30 treatment sessions, 4 days/week, except for patients 3 and 4, who received only 19 and 12 treatment sessions (due to health problems), respectively.

**Figure 7:**
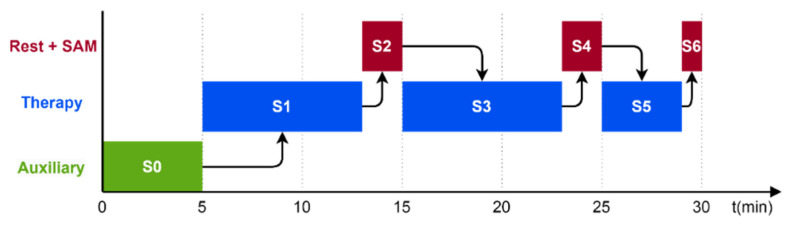
Temporal diagram of the protocol with the sequence of stages and their durations.

Each patient performed the SAM test three times (SAM1, SAM2 and SAM3 in Table 4) during each therapy session: once at stage 2 (after hand flexion/extension movements and precision grip) and also during stage 4 (after individual finger flexion/extension) and once again at stage 6 (after hand flexion/extension movements to squeeze oranges), as indicated in Figure 7.

### 2.5. Statistical Analysis

Statistical analyses were performed using the R Statistical Software [36], with the alpha level set to 0.05 for statistical significance. The scores coming from the SAM tests were analyzed: total mean score, mean score of each patient and mean score of all patients over the sessions. A mixed model analysis of variance (ANOVA) was performed to determine the influence that the explanatory variables had on the score of the SAM tests. The model includes two fixed explanatory variables (phase and test stage) and a random effect variable (patient). The test stages are SAM1, SAM2 and SAM3 tests, and the rehabilitation phase is the initial phase (1–10 days), the middle phase (11–20 days) and the final phase (21–30 days). Tukey’s HSD post hoc test is conducted to perform pairwise comparisons between test stages (SAM1, SAM2 and SAM3) and rehabilitation phases (initial, middle and final).

## 3. Results

**Figure 8:**
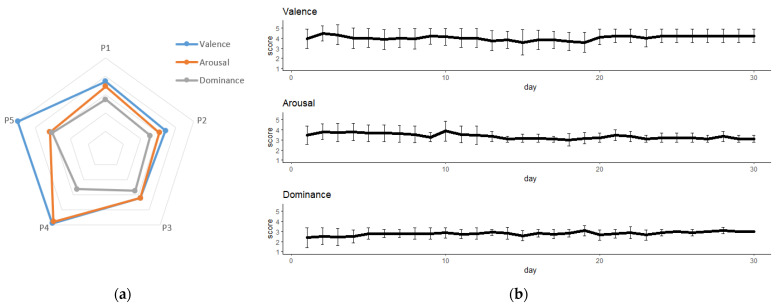
SAM test results: (**a**) mean score of each patient; (**b**) mean score of all patients.

The mean and standard deviations of valence, arousal and dominance obtained from all data gathered from the SAM evaluation were 3.2 ± 0.7, 2.60 ± 0.8 and 2.8 ± 0.2, respectively. The mean scores of each patient for valence, arousal and dominance are shown in Figure 8a. The evolution of the mean scores of valence, arousal and dominance over the 30 rehabilitation sessions is shown in Figure 8b.

The SAM tests scores were analyzed through Multifactorial Additive ANOVA. Numerical results extracted from this analysis are reported in Table 5, including *p*-values of the *F*-test. Significant differences are found in the two explanatory factors (phase and test stage) for the three emotional dimensions.

**Figure 9:**
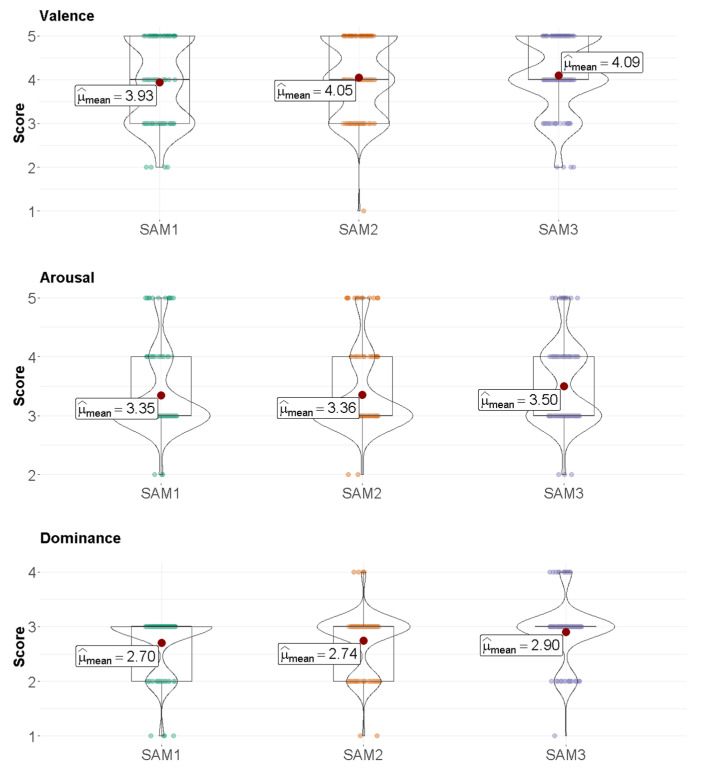
Boxplot of the results for the SAM1, SAM2 and SAM3 tests responses.

Numerical and graphical results extracted from the Tukey’s HSD post hoc test to compare test stages are reported in Table 6 and Figure 9. Specifically, Table 6 contains the two tests that are compared, the significance at the corrected level, the difference between the estimated group means, the lower and upper limits for the 95% confidence intervals of the true difference of means and the *p*-value for a hypothesis that the true difference of means for the corresponding groups is equal to zero. The distributions of the score results of SAM1, SAM2 and SAM3 are shown in Figure 9.

**Figure 10:**
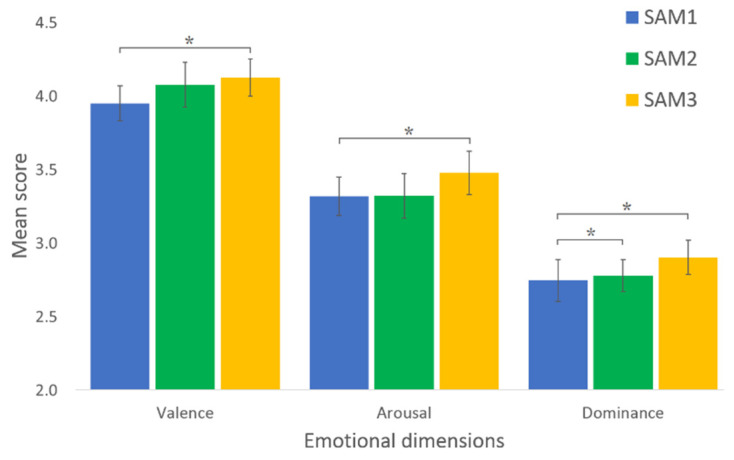
Score comparison between SAM1, SAM2 and SAM3 tests of the three emotional dimensions. * denotes significance at the (<0.05) level.

Figure 10 shows a graphical summary (mean score, standard deviation and significant differences) of the score comparison between SAM1, SAM2 and SAM3 tests of the three emotional dimensions. The statistical significance of the differences between the means of SAM1 and SAM3 tests are found in the case of valence, arousal and dominance. A significant difference is also found in the case of SAM1-SAM2 for dominance.

**Figure 11:**
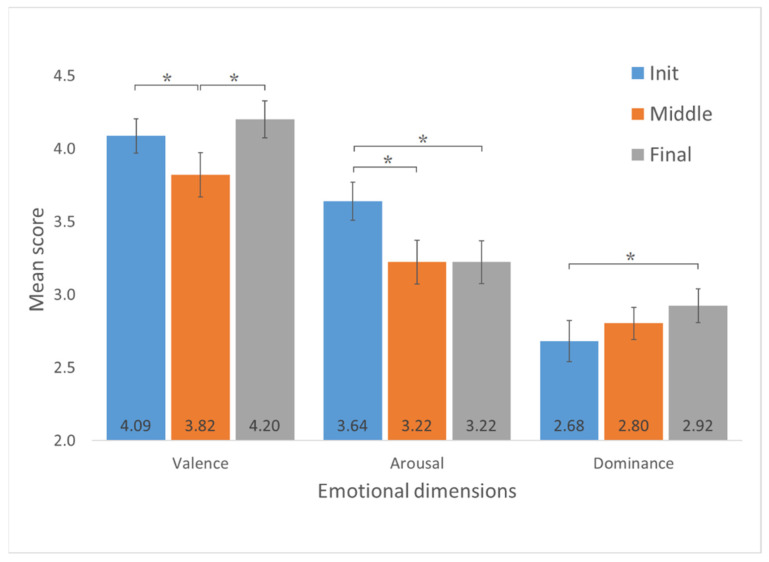
Score comparison between initial, middle and final rehabilitation stages for the three emotional dimensions. * denotes significance at the (<0.05) level.

With respect to the Tukey’s HSD post hoc test to compare rehabilitation phases, numerical and graphical results are reported in Table 7 and Figure 11. In the case of valence, the analysis shows the statistical significance of the differences between the means of the initial and middle phase and the means of the middle phase and final phase. In the case of arousal, the statistical significance of differences is between the means of the initial and middle phases and the means of the initial and final phases. In the case of dominance, the statistical significance of the differences is only between the initial and final phases.

## 4. Discussion

From the results obtained from the Multifactorial Additive ANOVA, it can be concluded that rehabilitation phase (initial, middle and final phases) and test stage (SAM1, SAM2 and SAM3 tests) statistically influence the three emotional dimensions (arousal, valence and dominance). The patients became familiar with the RobHand platform during the 30 rehabilitation sessions. The results extracted from the post hoc test to compare rehabilitation phases support this idea. First, the average dominance for the five patients increased during the sessions. In fact, there was a significant difference between the initial and final phase. This growth in dominance implies that the patients, at the end of the treatment, had a greater feeling of control over the rehabilitation tasks. With respect to the arousal, the mean scores decreased over the sessions. This decrease was significant over the initial phase and both the middle and final phases. This decline could be a consequence of the fact that when patients were becoming familiar with the robotic system, they found it less attractive because it was not a novelty anymore. Although the mean of the valence values during the first sessions was high, these values suffered a significant decrease in the middle phase and grew again, reaching higher levels than the initial ones during the last phase. No clear conclusions can be drawn from the results of the valence emotional dimension.

Significant differences were found between the results of the SAM1, SAM2 and SAM3 tests on the three evaluated emotional dimensions: valence, arousal and dominance. Both valence and arousal showed a significant difference from SAM1 to SAM3. The SAM3 test was performed right after the squeeze orange therapy T4, which is the only therapy with a realistic scenario, as it represents an everyday activity. This increase in both valence and arousal indicate that the patients felt happier and more stimulated performing T4. These results are consistent with many studies which claim that patients found rehabilitation therapies based on videogames more exciting [9,37,38]. There were also significant differences on the dominance parameter between the beginning (SAM1), the middle (SAM2) and the end of the therapy (SAM3). Thus, it can be concluded that the patients had a greater perception of controlling the robotic device over the duration of the rehabilitation session.

**Figure 12:**
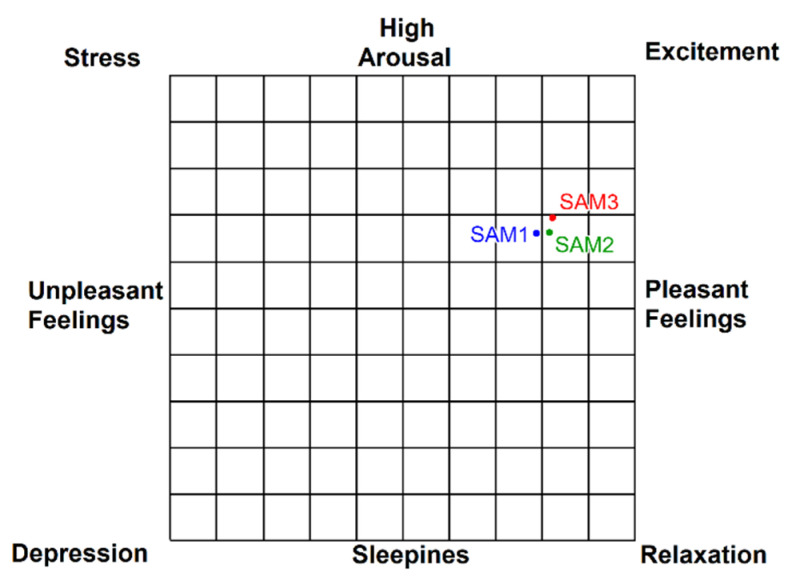
Representation of the mean arousal and valence scores using the Affect Grid.

Although emotions are elusive, the circumplex model of affect is a theory that claims that all human emotions can be described as a linear combination of two independent basic emotions: valence and arousal. Valence expresses whether the emotion is pleasant or unpleasant, and arousal determines how much the emotion is activated [18,39]. Following this idea, J.A. Russell also designed the affect grid, which is a single-item scale of pleasure and arousal [26]. Mean pleasure and arousal scores obtained in SAM1, SAM2 and SAM3 are plotted in this two-dimensional affective space (Figure 12). Both arousal and valence suffered an increase from SAM1 to SAM2, and also from SAM2 to SAM3, which, according to Russell’s model, means that the patients are more satisfied and relaxed over the duration of the therapy.

There are some limitations in the current study. The first one is related to the sample size since only five patients were included. The second one is related to the subjectively self-reported data, although it is an intrinsic risk in this type of study. Another limitation is that the subject may not clearly understand what is being asked about their emotional state. However, we chose the SAM questionnaire for this study because it was specially designed to minimize this risk as much as possible by using schematic pictures. Lastly, the order of the therapies (T1–T4) may influence the results. Since results with the robotic platform have been positive, further investigation is required with a larger sample size and considering the possible effect of therapy order.

## 5. Conclusions

The conducted case series study aimed to assess, using the SAM test, the emotional response of patients under rehabilitation using the RobHand platform. The outcomes are encouraging: the scores of arousal, valence and dominance are positive. It was verified that the emotions induced by the RobHand platform were successfully distributed in the three-dimensional emotional space. Over the 30-session treatment, a significant increase in dominance was found, coupled with a significant reduction in valence. Additionally, significant differences were found in the three emotional dimensions, indicating a positive variation when performing SAM3 since the therapy was carried out just after one based on a virtual environment, in which the user has the objective of squeezing oranges. Furthermore, the healthcare professional responsible for conducting and supervising this study subjectively detected that patients were much more excited when they performed the squeeze oranges therapy. The outcomes of the study encourage further development of videogame-based therapies in order to increase the patient’s positive feeling.

## Figures and Tables

**Table 1:** Adjective pairs associated with valence, arousal and dominance dimensions.

Dimension	Adjective Pairs
Valence	Unhappy–Happy Annoyed–Pleased Unsatisfied–Satisfied
Arousal	Relaxed–Stimulated Calm–Excited Sluggish–Frenzied
Dominance	Controlled–Controlling Influenced–Influential Cared for–In control

**Table 2:** Demographic and clinical characteristics of patients.

Patient	Sex	Age	Laterality Diagnosis
P1	male	61	Left Ischemic stroke
P2	male	43	Left Ischemic stroke
P3	male	22	Right Ischemic stroke
P4	female	38	Right Ischemic stroke
P5	male	53	Right Ischemic stroke

**Table 3:** Relationship between therapies, movement-based exercises and their virtual environment.

Therapies	Movement-Based Exercises	Virtual Environment
(T1)	(a)	Virtual hand only
(T2)	(c)	Virtual hand only
(T3)	(b)	Virtual hand only
(T4)	(a)	Virtual kitchen

**Table 4:** Protocol for patients performing therapy sessions with RobHand.

	Action	Duration
Stage 0 (S0)	Placement of exoskeleton on the patients’ hand	5 min
Stage 1 (S1)	Hand flexion/extension therapy (T1) and precision grips (T2)	8 min
Stage 2 (S2)	Rest break and SAM test (SAM1)	2 min
Stage 3 (S3)	Individual finger flexion/extension therapy (T3)	8 min
Stage 4 (S4)	Rest break and SAM test (SAM2)	2 min
Stage 5 (S5)	Squeeze oranges therapy (T4)	4 min
Stage 6 (S6)	SAM test (SAM3)	1 min
	Total	30 min

**Table 5:** Results extracted from a Multifactorial Additive ANOVA.

	Df	Sum Sq.	Mean Sq.	*F* Value	Pr (>F)	Significance
**Valence**						
Phase	2	3.56	1.7812	7.220	0.000845	*
Test Stage	2	1.60	0.8017	3.249	0.039960	*
Residuals	354	87.34	0.2467			
**Arousal**						
Phase	2	2.01	1.0028	4.409	0.0128	*
Test Stage	2	1.69	0.8457	3.718	0.0252	*
Residuals	354	80.52	0.2274			
**Dominance**						
Phase	2	2.99	1.4942	5.892	0.00304	*
Test Stage	2	2.65	1.3251	5.225	0.00580	*
Residuals	354	89.78	0.2536			

* denotes significance at the (<0.05) level.

**Table 6:** Results from the SAM tests showing the significant differences between test stages.

	Significance	Difference	Lower Limit	Upper Limit	*p*-Value
**Valence**					
SAM1–SAM2		0.11570248	−0.034488475	0.2658934	0.1666449
SAM1–SAM3	*	0.15702479	0.006833839	0.3072157	0.0380506
SAM2–SAM3		0.04132231	−0.108868641	0.1915133	0.7937554
**Arousal**					
SAM1–SAM2		0.008264463	−0.135800455	0.1523294	0.9899934
SAM1–SAM3	*	0.148760331	0.004695413	0.2928252	0.0411630
SAM2–SAM3		0.140495868	−0.003569050	0.2845608	0.0577416
**Dominance**					
SAM1–SAM2		0.04132231	−0.110641128	0.1932858	0.7980122
SAM1–SAM3	*	0.19834711	0.046383666	0.3503105	0.0064807
SAM2–SAM3	*	0.15702479	0.005061352	0.3089882	0.0409853

* denotes significance at the (<0.05) level.

**Table 7:** Results from the SAM tests showing the significant differences between rehabilitation phases.

	Significance	Difference	Lower Limit	Upper Limit	*p*-Value
**Valence**					
Initial–Middle	*	−0.2655285	−0.40773323	−0.1233237	0.0000436
Initial–Final		0.1133333	−0.04253927	0.2692059	0.2023516
Middle–Final	*	0.3788618	0.21670056	0.5410230	0.0000002
**Arousal**					
Initial–Middle	*	−0.40422764	−0.5407669	−0.2676884	0.0000000
Initial–Final	*	−0.41777778	−0.5674403	−0.2681153	0.0000000
Middle–Final		−0.01355014	−0.1692507	0.1421505	0.9771383
**Dominance**					
Initial– Middle		0.1248780	−0.01930057	0.2690567	0.1045848
Initial–Final	*	0.2422222	0.08418607	0.4002584	0.0010288
Middle–Final		0.1173442	−0.04706790	0.2817562	0.2143593

* denotes significance at the (<0.05) level.

## Data Availability

The data presented in this study are available on request from the corresponding author.
